# Procedural Memory Consolidation in Attention-Deficit/Hyperactivity Disorder Is Promoted by Scheduling of Practice to Evening Hours

**DOI:** 10.3389/fpsyt.2017.00140

**Published:** 2017-08-03

**Authors:** Maria Korman, Ishay Levy, Avi Karni

**Affiliations:** ^1^Faculty of Social Welfare and Health Sciences, Department of Occupational Therapy, University of Haifa, Haifa, Israel; ^2^E. J. Safra Brain Research Center for the Study of Learning Disabilities, Haifa, Israel; ^3^Laboratory for Functional Brain Imaging and Learning Research, Sagol Department of Neurobiology, University of Haifa, Haifa, Israel

**Keywords:** procedural learning, motor sequence, consolidation, attention-deficit/hyperactivity disorder, chronotype, evening training, young adults, training schedule

## Abstract

In young adults without attention-deficit/hyperactivity disorder (ADHD) training on a novel movement sequence results not only in large within-session (online) gains in task performance but also in additional (delayed, off-line) gains in the performance, expressed after an interval of sleep. In contrast, young people with ADHD, given an identical practice, were shown to improve online but expressed much smaller delayed gains overnight. As delayed gains in performance are taken to reflect procedural (“how to”) memory consolidation processes, this may explain skill learning deficits in persons with ADHD. However, motor training is usually provided in morning sessions, and, given that persons with ADHD are often evening types, chronobiological constraints may constitute a hidden factor. Here, we tested the hypothesis that evening training, compared to morning training, would result in larger overnight consolidation gains following practice on a novel motor task in young women with ADHD. Participants with (*N* = 25) and without (*N* = 24) ADHD were given training on a finger opposition sequence tapping task, either in the morning or at evening. Performance was assessed before and immediately after training, overnight, and at 2 weeks post-training. Individuals with ADHD reported a general preference for evening hours. Evening training was equally effective in participants with and without ADHD, both groups showing robust consolidation gains in task performance overnight. However, the ability to express delayed gains overnight was significantly reduced in participants with ADHD if trained in the morning. Typical peers were as effective in expressing overnight consolidation phase gains irrespective of the time-of-day wherein the training session was afforded. Nevertheless, even after morning training, participants with ADHD fully retained the gains acquired within the first 24 h over an interval of about 2 weeks. Our results suggest that procedural memory consolidation processes are extant and effective in ADHD, but require that specific biobehavioral conditions be met. The affordance of training in the evening hours can relax some of the constraints on these processes in ADHD. The current results are in line with the notion that the control of what is to be retained in procedural memory is atypical or more stringent in ADHD.

## Introduction

### Motor Learning in Healthy Adults

Procedural memory processes (1–4) subserve the mastering and retention of motor skills and have been characterized in typical adults by a distinct time course (5–8). Large improvements in speed of motor performance occur early on during training on a novel motor task, with no costs of accuracy (i.e., within-session gains, “fast” learning phase). Performance within-session reaches an asymptote as practice accumulates [e.g., Ref. (5)]. However, within several hours after the end of the training session, additional robust gains in performance can be expressed (as delayed, “off-line” gains), reflected in improved speed and accuracy of task performance, as well as in a reduction in performance variance (9–11). The off-line gains are considered to constitute a behavioral marker for the successful accomplishment of procedural memory consolidation processes, which are initiated by the training experience but requiring hours to evolve; processes whereby the neuronal substrates engaged during practice are changed according to the accrued experience (3, 12–15). The level of motor performance attained at the consolidation phase is typically retained over a period of weeks and even months in young adults (3, 6, 7, 16). In the FOS task, the task used in the current study, these gains were correlated with experience dependent motor cortex activity pattern changes (17).

Training-related factors such as the number of task repetitions and instruction are critical in determining the effectiveness and course of skill learning (10, 18–20). However, after the termination of the training experience, conflicting experience, or the availability of a post-training sleep interval can also critically affect the course of learning a new skill, specifically, by interacting with the consolidation processes (8, 14, 21). Sleep has been identified as a state wherein the consolidation of newly acquired information in memory is promoted, depending on the specific conditions of training, instruction, and proximity to sleep episode (22, 23). Sleep supports both quantitative and qualitative changes of memory representations (5, 8, 15, 24–27), and age- or health-related changes in sleep architecture were shown to disrupt normal consolidation processes (23). In typical young adults training in the FOS task, memory consolidation processes (as reflected in the expression of delayed, off-line, gains in performance, but also in a decreased susceptibility to interference by subsequent conflicting experiences) were shown to be accelerated and successfully completed not only by a night’s sleep but also by relatively shorter daytime naps (8). Thus, in the FOS task [with training in the standard protocol (5, 7, 8)], the evidence clearly indicates that rather than time *per se*, time in sleep is the critical factor gating the successful completion of consolidation processes, in both young adults and the elderly (28) [but not in preadolescent children (29)].

### Attention-Deficit/Hyperactivity Disorder (ADHD) Condition and Motor Learning Deficits in ADHD

Attention-deficit disorder (ADD)/ADHD is a neurological condition characterized by inattention and/or hyperactivity–impulsivity that interferes with everyday functioning. While attention problems are recognized as a core deficit (30), deficits in executive functioning (e.g., fluency, planning, inhibition, and set-shifting) (31) and motor functioning (32–34) are recognized as key characteristics.

Some theoretical accounts implicate deficits in procedural memory (skill acquisition) as a central deficit in ADHD (35). Findings from structural and functional neuroimaging studies of brains of individuals with ADHD have revealed differences compared to typical peers in multiple brain systems including circuits implicated in repeated task performance and skill learning (36–39). It is not clear, however, whether these differences relate to a less effective acquisition, or deficient consolidation or retention processes in ADHD. A simple view of ADHD as a procedural memory deficit may need to be qualified. The evidence from studies of implicit (SRT task) and explicit learning of movement sequences in adults and children with ADHD is equivocal; some studies report deficits vis-à-vis typical controls (32, 33, 40) while in other task conditions, participants with ADHD were as effective learners as their typical peers (40, 41). Deficits in the sustained engagement of attention resources and reduced inhibition of incorrect responses (42) were proposed as important factors leading to ineffective learning in ADHD.

A number of studies wherein the FOS task was used as the to-be-learned task suggest that young adults with ADHD may exhibit an atypical procedural memory consolidation process rather than a critical deficit (32, 43, 44). For example, in a study that compared the time course of learning following the FOS training in young females with and without ADHD, the ADHD group exhibited normal within-session gains in performance speed, but the delayed gains measured at the 24 h post-training retest, were reduced (32). Given the pivotal role of procedural memory for everyday functioning (e.g., the skill of driving) (3, 4), it would be reasonable to expect that short-term motor deficiencies occurring in the individuals with ADHD would have to be at least partially compensated on the long run. Indeed, the study by Adi-Japha and her colleagues (32) demonstrated that the relative initial deficits in the expression of overnight delayed gains turned out to be temporary; the performance gap vis-à-vis typical peers training in an identical protocol diminished within a few days. The real potential of the individuals with ADHD to acquire novel skills may, therefore, be uncovered by manipulating factors that gate brain plasticity; for example, more stringent demands may be set on factors such as attention and arousal levels or post-training sleep quality and timing in ADHD. Indeed, some of the relative learning deficits, in persons with ADHD, could be corrected when training was shortened (32, 33, 44, 45) perhaps decreasing the burden of long repetitive practice on mechanisms of sustained attention (46). The upregulation of arousal levels, which are typically low in ADHD (47), for example, by the means of whole body vibration (48) and white noise (49) stimulation, has been shown to enhance both attention and motor performance.

In typically developing adults, sleep after practicing a new motor skill supports memory consolidation processes, contributing to the generation of stable, enhanced and long lasting procedural memory representations (5, 7, 50–52). A comorbidity of ADHD and sleep disorders is recognized; over 65% of individuals with ADHD may present with one or more sleep disorders (53–56). A recent review suggests that sleep problems and ADHD interact in a complex bidirectional manner with sleep disturbances exacerbating or exacerbated by ADHD symptoms (53).

### Chronotype, Sleep, and Learning in ADHD

Chronotype is an individual characteristic reflecting the time of day at which a person is “at his best” (57); “eveningness” (delayed sleep period, alertness reaches the maximum values at 11 p.m.) and “morningness” (advanced sleep period, alertness reaches the maximum at 8 a.m.) are the two extremes with most individuals in the general healthy population preferring the period between these extremes (58, 59).

Adult ADHD is associated with the evening chronotype (60–62). More than 40% of adults with ADHD display an evening preference; in age-matched healthy peers in general population, only 10.8% exhibit evening preference (63). Morning preference is switched, respectively, 40.2% in the typical population and 18.5% in ADHD. Greater eveningness correlates with the core symptoms of inattention and increased impulsivity; as eveningness is associated with shorter night sleep period, sleep debt may play a causal role in these symptoms (63). Additional evidence for a link between ADHD symptoms and circadian disruption comes from findings that seasonal affective disorder, a depression disorder directly linked to circadian disruption shows high comorbidity with ADHD (55, 64, 65). It was also suggested that the hyperactivity of people with ADHD may lead to sleep deprivation (66). The core symptoms of ADHD, such as inattention, impulsivity, and impatience, are typical outcomes of sleep deprivation in typical adults (67).

Consistent failure to meet basic sleep needs is currently viewed as a significant contributor to the cognitive and behavioral deficits in individuals with ADHD (53). As many as 70% of children and up to 83% of adults with ADHD have been reported as having sleep problems (68, 69) with sleep onset insomnia (SOI), the most common problem (54). A study that compared ADHD with and without SOI reported that 78% of adults with ADHD reported SOI, but when tested objectively by actigraphy, no difference in basic sleep parameters (duration and efficiency of sleep, as well as sleep onset latency) were found between those reporting or not reporting SOI (55). However, compared to typical controls, the participants with ADHD showed extended sleep onset latencies and lower sleep efficiency. Adults with ADHD also report reduced sleep quality, difficulty in getting to sleep, and difficulty in waking up (70). Individuals with ADHD were found to sleep on average an hour less than controls on nights prior to work days (but not prior to free days) and showed larger variability in bedtimes and sleep latencies (64). More than 60% of adults with ADHD reported increased sleepiness during day time (54, 55, 70). Delayed timing of melatonin secretion is systematically found in children and adults with ADHD (54, 55, 71). Rybak et al. suggested that a substantial circadian phase delay considerably impacts the core pathology of the ADHD (72).

Brain plasticity, the basis for skill and knowledge, is a slow and highly controlled (selective) process, wherein synaptic and cellular modifications occur at brain circuits in which the memory was initially encoded during salient experiences. Multiple lines of evidence suggest that these processes proceed “off-line,” during both wakefulness and sleep, and culminate in the consolidation of new information and it’s integration into previously existed knowledge (3, 5, 8, 15). Whether these off-line processes will be allowed to proceed to a successful completion is under strict control (“gating”) (73). Optimal arousal level during encoding is considered a prerequisite gating factor mediating the long-term memory formation (47). Memory systems (74) and cognitive processes such as attention and executive functions (75, 76), as well as reward processes (77–79), are sensitive to disruptions of sleep and circadian rhythms. Indeed, the circadian clock, the reward system, and memory processes directly or indirectly affect neurogenesis and neural growth and shaping processes (80). Light acts on all three systems through common basic signaling pathways (80) and all three are affected by the hypothalamic–pituitary–adrenal axis *via* cortisol (81). Moreover, the evidence that most of the genes that shape the biological clock are expressed in brain areas that are associated with learning, memory, and reward, such as the amygdala, the hippocampus, and the ventral tegmental areas, is in line with the notion that the endogenous ~24 h time-generator (suprachiasmatic nucleus) has a role in gating neuronal plasticity following daily experiences (81).

### The Current Study

The majority of training protocols used in memory research afford training sessions during morning or early afternoon, a time of day that may be suboptimal for individuals with evening chronotype and/or with higher susceptibility to interference [like the persons with ADHD (55) or the elderly (28)]. Recently, it was proposed that post-training sleep and its timing relative to the training experience is a critical factor in the control (gating) of motor skill memory selectivity in young adolescents (82) and in the elderly (28). Similar constrains may be imposed on mnemonic processes in individuals with ADHD during the morning hours so as to limit the generation of long-term memory from experiences gained in less than optimal practice-learning conditions; i.e., when alertness and cognitive abilities are at the diurnal minimum (83). Thus, memory deficits may be, at least in part, a result of the timing of the training experience rather than a general deficit in motor skill consolidation. In the current study, we tested the hypothesis that practice in the evening hours compared to morning hours may provide better conditions for the engagement of, the presumably atypical, consolidation processes in young women with ADHD. Operationally, we expected that training in the evening hours will result in higher delayed, overnight, gains in performance than training in the morning in the ADHD groups. In contrast, we expected that in the control groups (typical adults), delayed gains in performance will evolve regardless of the timing of training session.

We chose to address in the current study only young women with ADHD because (i) the performance of skilled movements in ADHD were suggested to be gender dependent (84, 85), (ii) between individual variances in the symptomatology of ADHD is smaller in females compared to males (86) and thus a smaller number of participants can be used in an exploratory study, and (iii) to enable a direct comparison to the results of previous studies (32, 44) wherein consolidation processes were systematically explored in young women with ADHD, using the FOS task (the task used in the current study).

## Materials and Methods

The study was approved by the Human Experimentation Ethics committee of the University of Haifa and the participants signed an informed consent form in accordance with the Declaration of Helsinki before beginning the experiments. Subjects were paid 150 shekels (approximately $37) for their participation.

### Participants

Forty-nine right-handed (87) young (age between 20 and 35 years) females, University of Haifa students, enrolled in the study. Participants were recruited through advertisement boards at the University of Haifa and the University center for students with disabilities, for a “motor learning and memory study.” 24 participants met the criteria for a DSM-IV diagnosis of ADHD, and 25 typically developing adults matched by age and education, served as a control group. Inclusion criteria for the ADHD groups were as follows: (1) a formal psycho-didactic diagnosis of an attention-deficit disorder (either ADD or ADHD) from an authorized clinician, psychiatrist, or neurologist, approved by the University center for students with disabilities within 5 years of the current study; (2) a positive screening on the adult ADHD self-report scale (ASRS) (88, 89); and (3) no stimulant treatment for ADHD (methylphenidate or other stimulant drugs) during the recent period (> month). The participants of the ADHD group had on average 11 out of 18 items (10.9 ± 2.7, mean ± SD) positive responses on the ASRS. The control participants met less than 3 out of 6 criteria of the ASRS screening questionnaire (first 6 items). All control participants affirmed that they were not suggested (by family members or teachers) to have or were never diagnosed as having ADHD/ADD during their childhood or adulthood.

All participants underwent a semi-structured interview to exclude persons with diagnosed sleep, neurological or psychiatric disorders, motor-skeletal diseases, and use of chronic medications or drugs. Four of the participants with ADHD, but none of the participants in the control group, reported that they were previously diagnosed as having dyslexia. All participants underwent chronotype assessment using the Horne–Östberg Morningness–Eveningness Questionnaire (MEQ) (90). MEQ assesses whether a person’s peak alertness is in the morning, in the evening, or in between. Higher sum scores are associated with morningness, while lower scores point to eveningness. The MEQ is a widely used and reliable scale to measure circadian type (91, 92).

Participants reporting skilled “blind” typing or professional string instrument playing and those reporting sleeping less than 6 h per night routinely, were excluded (5, 32, 43). The participants were instructed not to practice the study task that they were trained on between the scheduled meetings and not to drink caffeine containing drinks during the experiment.

### Task and Procedure

**Figure 1:**
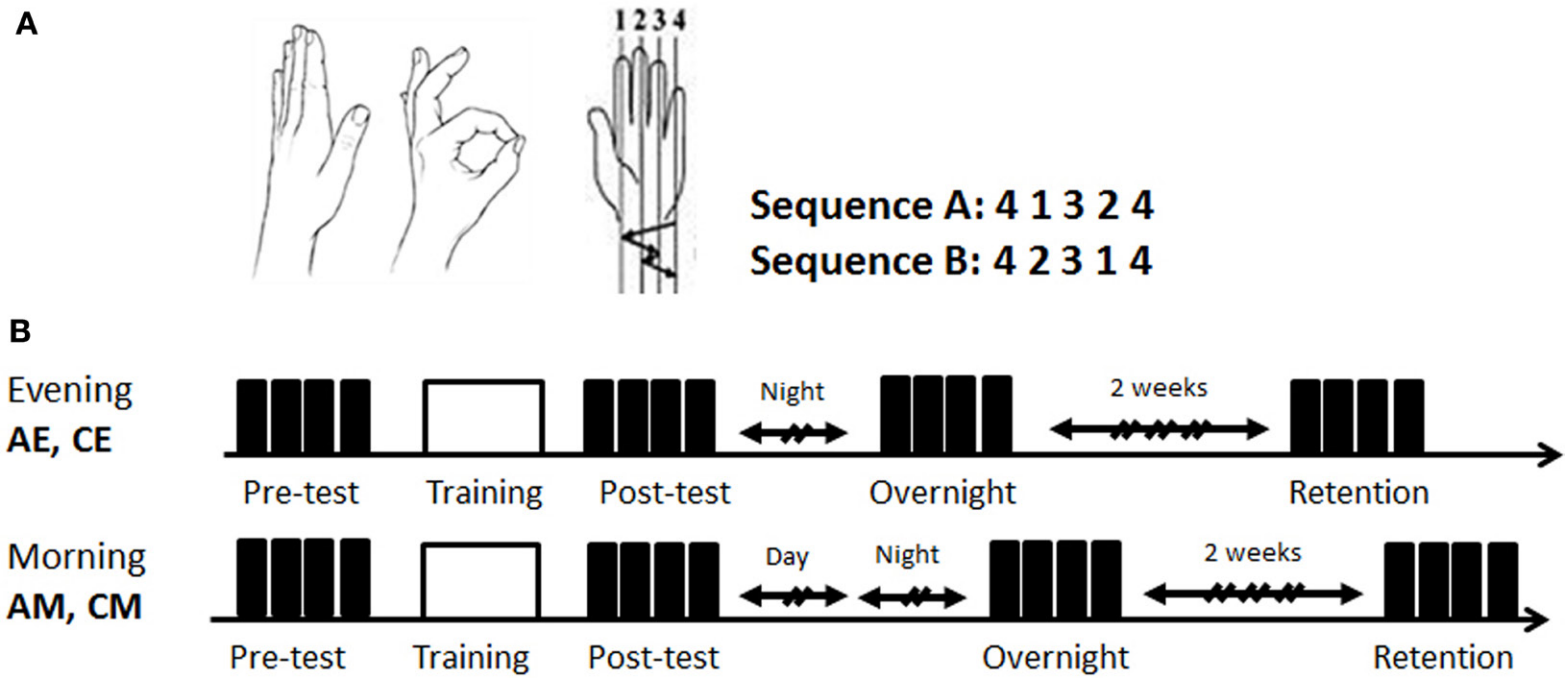
Task and study design. **(A)** The finger-to-thumb opposition task. The two 5-element finger-to-thumb opposition sequences were mirror reversed order of movements to each other. Each participant was randomly assigned one of the sequences (A or B); **(B)** the time line of the two experimental conditions. All participants were trained in an identical single session (160 cued repetitions of the sequence) (white box) afforded either at 7:00 p.m. to 9 p.m. (evening) or at 8:00 a.m. to 10:30 a.m. (morning). Both the evening and the morning groups were retested in the morning hours of the next day. Performance was tested at four time-points: pretest, posttest, overnight, and 2 weeks retention post-training (black boxes—test blocks; h—hours). EA, ADHD training at evening; EC, typical controls trained at evening; MA, ADHD trained at morning; MC, typical controls trained at morning.

The participants were trained and tested in performing an explicitly instructed five-element finger-to-thumb opposition sequence (Figure 1A) (5, 32). All tests were performed with the participants sited in the arm-chair with their left (task performing) arm positioned, comfortably extended, with the palm facing up to allow video recording of all finger movements. Visual feedback was not allowed; the participants were instructed to avert the gaze away from the fingers of the performing hand.

The experiment included three sessions. In the first session, lasting approximately 30 min, the experimenter showed the thumb-to-finger opposition movements, without demonstration of the sequence. Participants received verbal instructions, informed which sequence they were assigned to (randomly chosen A or B, Figure 1A) and performed self-paced warm-up sequences three times. Following the correct performance of the three warm-up trials, a pre-training performance test, a training, and a post-training performance test were afforded. A performance test consisted of four self-paced blocks, each 30 s long. An explicit instruction was provided before each block to perform the assigned sequence of finger movements “as fast and as accurate as possible” between the start and the stop sounds, given by the computer. Occasional errors should not be corrected. A 30 s rest interval was afforded between the test blocks. Following completion of the four test blocks (pretest), the participants performed 20, 30-s, cued training blocks (training), with a 30-s rest between blocks, altogether 160 repetitions of the sequence. Rest periods could be prolonged if requested by the participant.

Start of each sequence was signaled by a beep at a rate 2.5 s per sequence. No feedback was provided on correctness and speed of performance. Following the training, participants again performed four test blocks (posttest), with identical instructions to the initial tests.

The participants were randomly assigned to four groups (Figure 1B): two groups, ADHD-morning (AM, *n* = 12) and control morning (CM, *n* = 13) were trained in the morning (8:00 a.m. to 10:30 a.m.); and two additional groups, ADHD-evening (AE, *n* = 12) and control evening (CE, *n* = 12), received an identical training session in the evening (7:00 a.m. to 9:00 p.m.) on the first day of the experiment. The first session (first day, evening, or morning) included the baseline performance test on the assigned sequence (pretest), the training session and the post-test. All participants were retested during second session in the morning of the next day (overnight retest, 5 min long). The third, retention test (third day session, 15 min long), took place on average 14 days (±2) after the second session and was performed again during morning hours (8:00 a.m. to 10:30 a.m.).

Participants were asked to wear an actiwatch (Actigraph Co.) starting from the end of the immediate post-training test to the next 5–7 days, so as to record sleep times and quality. Actigraphy was optional; a consent to wear an actiwatch did not constitute an inclusion criterion. The data were analyzed using ActiLife 6 software.

### Statistical Analysis

Performance data were analyzed off-line in terms of speed (number of correct sequences) and accuracy (number of errors) performed per test block from video recordings. Average speed and accuracy of the four test-blocks at each of the four time points (pre-training; post-training; 12–24 h post-training; retention) was calculated. Speed and accuracy of performance were analyzed separately using: (a) a repeated measures analysis of variance with the four time points as within-subject factors × 4 groups [ADHD morning (AM); ADHD evening (AE); CM; CE] as a between subjects factor; and (b) a repeated measures analysis of variance with two consecutive time points to test performance changes across different stages of learning: acquisition phase—fast learning (pre-training vs. post-training), consolidation phase—slow learning (post-training vs. 24 h post-training) and retention phase (24 h post-training vs. retention). Two-tailed *t*-tests corrected for multiple comparisons were used in the analysis of the normalized performance gains with level of significance of *p* < 0.05.

## Results

### Chronotype and Sleep Data

Mean group MEQ scores differed between persons with ADHD and healthy controls (two-sample *t*-test, *t* = −4.127, *p* < 0.001); lower scores, corresponding to larger eveningness were found for the ADHD group (Table 1). The proportion of participants expressing a certain chronotype was significantly different between the groups (χ = 9.17, *p* = 0.043; Fisher’s exact test, *p* = 0.048). More eveningness types were found in the ADHD group than in the control group (45.8 vs. 12%). Also, there was a significant difference between the ADHD and the control groups when the MEQ score (continuous measure) were compared (two-sample *t*-test, *t* = 4.290, *p* < 0.001). There was, however, no significant difference in the MEQ scores of the participants with ADHD who were trained in the morning as compared to those receiving evening training (two-sample *t*-test, *t* = 0.660, *p* = 0.516; EA group—5/12, MA group—6/13). Similarly, no significant difference in the MEQ scores of the control participants who were trained in the morning as compared to those trained in the evening was found (two-sample *t*-test, *t* = −0.510, *p* = 0.615; EC group—1/12, MC group—2/12).

**Table 1:** Morningness–eveningness questionnaire (MEQ) continuous and categorical scores for the ADHD and control participants.

Type	ADHD (*n* = 24)	Control (*n* = 25)
MEQ mean ± SE	42 ± 1.89	53.86 ± 2.03
Morning type	0	1
Moderately morning type	2	6
Neither type	11	15
Moderately evening type	7	3
Definitely evening type	4	0

*The continuous scores were translated into categorical chronotypes using standard cutoff criteria (90, 91)*.

As the participation in actigraphy was voluntary, the actigraphy data sample is limited and contains selected participants in each condition [ADHD *n* = 16, control *n* = 12; AM *n* = 7, AE *n* = 9, CM *n* = 6, CE *n* = 6]. Average time-in-bed, sleep latency (time to fall asleep), total sleep time (minutes), and sleep efficiency parameters, averaged across 5–7 nights starting from the first night following the training session, were analyzed using two-tailed independent sample *t*-tests. Results showed significant main effect of group (ADHD, control) for total sleep time (*t* = −2.722, *p* = 0.011), reflecting shorter night sleep in ADHD participants (ADHD: 400.18 ± 74 min, control: 498.46 ± 115 min). No significant differences were found with regard to sleep efficiency (mean 92.3 ± 8.6%), sleep latency (mean 4.3 ± 2.9 min) and time-in-bed. All participants reported a high subjective sleep quality during the experimental period. No significant correlations between chronotypes and sleep parameters and the observed gains in performance speed at the posttest, overnight, and retention test points were found.

### Behavioral Data

**Figure 2:**
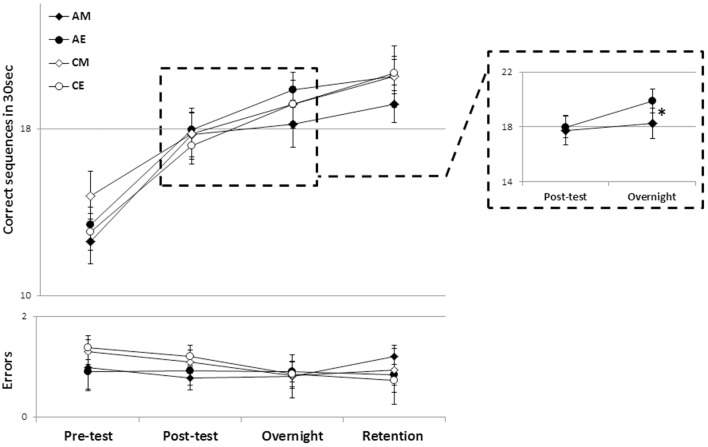
The time course of performance improvement in the four groups. There were clear within-session gains as well as delayed (post-training) “off-line” gains in speed that were well maintained across a 2-week retention interval (upper panel) with no costs in accuracy (lower panel). Each data point depicts the mean group performance for four time-points. Note that the overnight time-point denotes performance on the morning of the post-training day (~12 or 24 h post-training for the evening and morning training groups, respectively). AM, ADHD morning; AE, ADHD evening; CM, control morning; CE, control evening (bars, SEM). Inset: a magnified view of the AM and AE groups’ performance across the first overnight interval (consolidation phase). *Significant interaction effect.

First, we excluded the possibility of a confounding effect of pre-training differences in performance between the experimental groups. Independent samples, two-tailed *t*-tests showed that there were no significant difference between the pretest performance of the two control groups (CM, CE) (*p* = 0.12) as well as between the two ADHD groups (AM, AE) (*p* = 0.558). There were also no significant differences between the participants with ADHD and their corresponding control groups when tested in the morning (MA, MC; *p* = 0.22) or in the evening (EA, EC; *p* = 0.33). Thus, the baseline performance of all participants was not significantly affected by the time of test (morning or evening) or ADHD status (Figure 2).

Training on the assigned sequence of movements resulted in early (within-session) and delayed (post-training, time-dependent) gains in performance triggered by a single training session in all groups (Figure 2). An analysis of variance with repeated measures (rm-ANOVA) with four groups × 4 time points, showed that, overall, there was a significant improvement in speed [*F*(3, 43) = 78.46, *p* < 0.001] (Figure 2, upper panel) across the study period in all groups. There was no significant group effect (*p* = 0.705). There was, however, a trend toward a significant interaction of time-point × group [*F*(9, 135) = 1.78, *p* = 0.079] suggesting that the performance changes were dissimilar across the four groups.

On average, the participants in all four groups tended to commit, if any, very few errors (Figure 2, lower panel). Absolute accuracy did not change significantly across the period tested [*F*(3, 43) = 1.396, *p* = 0.247], suggesting that in all groups the improvements in speed were not at the cost of increased errors.

To explore which of the time intervals contributed to the trend toward an interaction of time-point and group, in performance speed, *post hoc* rm-ANOVA comparing pairs of consecutive time-points were conducted across the four groups. A significant interaction of time-point × group was found only for the post-session consolidation interval, i.e., in comparing between the posttest and the overnight post-training retest [*F*(3, 45) = 3.31, *p* = 0.028]; indicating a significant difference in the rate of performance improvement overnight in the different groups. As can be seen in Figure 2 (inset), the ADHD morning group lagged behind their peers who received the identical training protocol but in the evening, as well as behind the participants in the two control groups. To directly test the contribution of the time of training to the expression of overnight, delayed, gains in performance, in participants with ADHD, an rm-ANOVA was performed comparing the two time-points (posttest, overnight) in the two ADHD groups (AM, AE). Although there was no significant group effect (*p* = 0.49), there was a significant time-point effect [*F*(1, 22) = 22.24, *p* < 0.001] indicating overall gains, but also a significant interaction of time-point × group [*F*(1, 22) = 7.55, *p* = 0.012] reflecting the smaller gains in the ADHD morning group (Figure 2, inset). A similar analysis comparing the overnight, delayed gains in performance speed in the two Control groups showed a significant overall improvement in both groups (CM, CE) [*F*(1, 23) = 39.9, *p* < 0.001] but no significant group (i.e., time of day) effect (*p* = 0.58) as well as, importantly, no significant time-point × group interaction (*p* = 0.29) suggesting that both groups improved at a similar rate.

The time of day in which training was afforded had, however, no significant effect on the ability to retain the gains in speed across the 2 weeks interval (Figure 2). An rm-ANOVA comparing performance in the last two time-points (overnight, retention) in the four groups showed that, rather than forgetting, there was a significant improvement in speed across the retention interval [*F*(1, 45) = 12.26, *p* = 0.001], but no significant group effect (*p* = 0.788), reflecting the finding that the gap that opened between the AM groups performance and that of their peers (irrespective of ADHD status) did not close at 2 weeks post training. The relatively smaller speed gains of the AM group were as well retained as those of the other participant groups.

**Figure 3:**
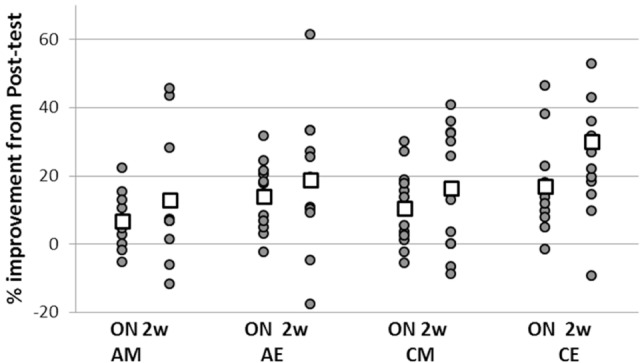
Individual normalized gains in task performance speed expressed at overnight and retention. Overnight gains (ON—the difference between overnight and posttest) were normalized to performance in the posttest; total gains (2 w—difference between retention and the immediate posttest) were normalized to pretest performance for each individual participant. Positive values indicate delayed gains in task performance; negative values correspond to a slowing down of performance speed relative to immediate post training levels. Squares—group averages.

Although there were no significant differences between the four groups’ average performance, there were large individual differences in pre-training performance, irrespective of ADHD status. To ensure that these large differences between individuals’ task performance levels did not bias the analyses based on absolute performance measures, we also assessed the differences in the expression of delayed gains in performance, with respect to the time of day training was afforded, using normalized data (Figure 3).

To this end, each participant’s gains in the overnight post-training interval (i.e., the difference between overnight and posttest) were normalized to pre-training performance. In addition, normalization to pre-training performance was done for the total post-training gains expressed in the retention test (i.e., for the difference between retention and the immediate posttest) (Figure 3). There was a significant difference between the two ADHD groups (AM, AE) in the overnight interval (two-sample *t*-test, *t* = −0.81, *p* = 0.042) reflecting an advantage for the evening group. In addition, the overnight performance gains of the ADHD morning group were significantly smaller compared to the control participants trained in the evening (two-sample *t*-test, *t* = −2.085, *p* = 0.05) though not significantly smaller than the gains of the CM group (*p* = 0.36). However, the overnight gains of the ADHD evening group (AE) were not significantly different from the gains attained by their typical peers trained either at morning (CM) or evening (CE) (*p* = 0.43, *p* = 0.54, respectively). There were no significant differences in the normalized performance gains expressed over the 2 weeks retention period in the four groups.

## Discussion

The present findings suggest that procedural memory consolidation processes are extant and effective in ADHD, but necessitate specific circadian conditions in order to be fully expressed. The current results, therefore, suggest a new effective learning strategy for ADHD. In line with previous studies (32), persons with ADHD showed the expected gains within the training session but less-than-expected performance gains, evolving overnight, during the procedural memory consolidation phase, if the training session took place in the morning hours. The same training experience afforded in the evening was equally effective in participants with and without ADHD, with both groups improving within-session as well as expressing additional, robust gains in task performance overnight. Nevertheless, morning training afforded to individuals with ADHD was as effective as evening training in terms of the ability to retain the gains acquired within the first 24 h post-training over an interval of about 2 weeks. Moreover, the retention of the training induced gains in performance was as effective in individuals with ADHD as in their typical peers with no ADHD.

Importantly, the current results show that the disadvantage of morning training for ADHD was not related to their ability to improve within session, regardless of the time of training. This result is in line with previous studies (32). However, the relative disadvantage of the morning trained individuals with ADHD was in their ability to express delayed, consolidation phase gains following their quite effective within-session learning. This relative performance lag was maintained over the retention interval.

### Chronotype and Sleep

There is good evidence supporting the notion that the affordance of an interval of sleep after a training experience constitutes an important factor in the expression of practice-dependent delayed (“off-line”) gains in the performance of the FOS task in young adults (5, 14, 93) and perhaps more so in elderly individuals (28). There are ample data suggesting that sleep structure may be atypical in persons with ADHD (54, 69, 94). In line with these notions, in the current study, individuals with ADHD tended to be evening chronotypes and to have on average shorter sleep durations. However, the robust overnight expression of delayed gains in the performance of the FOS, in persons with and without ADHD, after evening training, suggests that the post-training sleep intervals were equally sufficient in both groups in supporting the consolidation process.

The prevalence of late chronotypes among young adults in general population is less frequent than in those with ADHD but still significant, reaching 10–15% (95, 96); the low number of participants with evening chronotype in the control groups of the current study is in line with these reported frequencies. However, little is known about the contribution of chronotype to memory in healthy typical individuals. Future studies should address whether evening persons in the general population, those with no ADHD symptoms, may benefit from scheduling of learning session to evening hours, in analogy to the effects found for persons with ADHD. This is especially pertinent in adolescence, a phase of development wherein the circadian profiles are skewed toward eveningness (97).

### Procedural Memory Processes in ADHD

The current results provide support for several notions pertaining to skill memory processes in adults with ADHD. First, there is evidence suggesting that the acquisition and consolidation of a recently acquired memory trace, pertaining to a trained movement sequence, interact, but nevertheless constitute independent processes; each of these processes may require a different set of specific conditions to be effectively completed (15, 98, 99). Our results support this notion—young women with ADHD were as effective learners in the morning and evening hours as their typical peers, but they did differ in terms of their ability to subsequently (overnight) express consolidation phase gains. Thus, learning (acquisition, potentially reversible) and memory (dependent on consolidation) may differ from each other with regard to critically important control processes and gating factors. Proximity of evening training to sleep interval may be critical for successful engagement of consolidation processes for persons with ADHD. Not mutually exclusive is the possibility that, in the evening type persons, circadian factors affecting consolidation processes, for example, more effective synaptic tagging (100), are (also) at work.

A second notion is that while procedural memory mechanisms in young adults with ADHD may differ from those subserving skill consolidation in typical individuals, individuals with ADHD nevertheless can generate and effectively retain procedural memory. Atypical procedural memory consolidation processes in young adults with ADHD were indicated in previous studies of motor learning using the FOS task (32, 101). Nevertheless, in both studies, as well as in a study addressing FOS task learning and motor memory consolidation in adolescents with ADHD receiving methylphenidate treatment (43), there was clear evidence, despite atypical learning patterns, for effective long-term retention of skill in the individuals with ADHD. The current results, however, support the notion that young women with ADHD practicing the FOS task may differ from their typical peers in the conditions under which the engagement of consolidation processes occurs. Thus, young women with ADHD may atypically engage consolidation processes when trained in the morning, but not when trained in the evening.

A third notion concerns the training conditions. Conditions that are well suited for typical young adults may be less than optimal for individuals with ADHD. Thus, the apparent consolidation phase deficits in individuals with ADHD may reflect an interaction of the specific learning (and test) conditions with the individuals’ predispositions and chronotype, rather than the latter’s specific deficits *per se*. For example, Fox and colleagues (101) showed that halving (shortening) the training session may be beneficial for the training of persons with ADHD; perhaps because individuals with ADHD tend to commit more errors in tasks and tests that require multiple repetitions (33, 42, 45, 102). We extend this notion to account for time of training as an important condition, given that in people with ADHD show predominant chronotypes that are skewed toward eveningness. Optimal arousal level during encoding is considered to be a prerequisite, gating factor, mediating the process of long-term memory formation (47). Thus, the endogenous biological clock should be considered as gating factor to neuronal plasticity induced by daily experiences (81).

Altogether, we propose that consolidation processes are under stricter control in individuals with ADHD compared to their typically developing peers. A similar notion of extant procedural memory consolidation mechanisms that may be under stricter constraints compared to that of typical young adults has been recently suggested in explaining the findings in elderly individuals (28). Korman and her colleagues have shown that motor skill acquisition is well preserved in healthy elderly individuals, however, unless a post-training nap was afforded, overnight (consolidation phase, “off-line”) gains were under-expressed. The current findings indicate a similar pattern, with evening training critical for the expression of the full potential for overnight gains, in young women with ADHD. Thus, in analogy to the case of the healthy elderly, we propose that the apparent deficits observed after morning training in individuals with ADHD may reflect suboptimal engagement of procedural, “how to” memory consolidation processes rather than a core deficit in procedural memory consolidation abilities *per se* [as suggested for example by Nicolson and Fawcett (35)]. We do not suggest that the processes underlying the hypothesized under-engagement (or stricter control) of procedural memory processes in healthy elderly and in young adults with ADHD are identical. The proposal rather is that, in both populations, some added constraints are imposed on the selection of what is to be maintained in long-term memory after a given learning experience, compared to the constraints imposed on consolidation processes in typical young adults. Different constraints on consolidation processes (rather than differences in the capacity to learn or generate long-term procedural memory *per se*) have also been indicated by recent studies addressing developmental effects in FOS consolidation, i.e., before and after puberty in typically developing individuals (20, 29, 103).

### Limitations

Several considerations may limit the interpretation of our findings, given the different first retest periods across study groups trained in the morning and evening hours. Unequal time periods from training to subsequent testing may have contributed to processes of interference or enhancement (104), independently of circadian optimal time-windows for skill acquisition.

An increased susceptibility to interference (e.g., by everyday activities, following training session for which the new movement sequence is irrelevant) was suggested as a mechanism for applying a stricter consistency criterion on what is to be incorporated into long-term procedural memory (28, 105). One could suppose that, in adults with ADHD, there is an increased susceptibility to interference experiences during the waking hours after the training session, leading to smaller consolidation gains in performance. This possibility should be further investigated. However, a recent study suggested that overall susceptibility to interference by a subsequent conflicting experience is not enhanced, but rather is reduced in young women with ADHD (44), compared to typical peers.

The protocol using different delay periods across study groups trained in the morning and evening hours (all groups were retested the next morning) was implemented in to neutralize the possible differences in performance resulting from the time of post-training testing. Thus, the morning groups had more time (~24 h) to consolidate the newly acquired knowledge compared to the evening groups (~13 h). If time *per se* would be the critical factor to determine the amount of the delayed gains in performance, one would expect to find different levels of overnight performance in the control (evening and morning) groups. However, our findings clearly indicate that this is not the case. As well, in order to control for the confounding effect of the different time-periods following training, we have tested for skill retention at 2-week post-training, allowing ample time to complete the memory consolidation process. The results clearly indicate that: (1) all groups show robust retention (thus no forgetting) and, in fact, additional gains compared to the performance at the first retest; (2) the morning trained ADHD group still lags behind. Thus, our data show no forgetting in time intervals as long as 12–14 days post-training, and support the main interpretation of our results, of the disadvantage of morning training in young adults with ADHD.

Further, we note that current results are limited to a population of highly functional young females with and without ADHD (university students). Additional studies should be conducted in males or mixed experimental groups and in different age groups to afford more general conclusions.

## Conclusion

The current study provides evidence to suggest that in individuals with ADHD (frequently exhibiting evening chronotypes), training session afforded during morning hours negatively affect procedural memory consolidation (off-line, delayed) processes. Thus, individuals with ADHD may benefit from training protocols that have been optimized for their own advantage rather than from protocols optimized for their typical peers. Just as the length of the training session (101) or the spacing (rest periods) within and between practice sessions (106, 107) need to be taken into consideration when adapting training protocols for the benefit of persons with ADHD, an adjustment of the diurnal scheduling of the training protocol may be necessary for the full expression of the potential for skill acquisition and its consolidation in persons with ADHD.

## Ethics Statement

The study was approved by the Human Experimentation Ethics committee of the University of Haifa and the participants signed an informed consent form in accordance with the Declaration of Helsinki before beginning the experiments. Subjects were paid 150 shekels (approximately $37) for their participation.

## Author Contributions

MK, IL, and AK conceived and designed the experiments. IL collected the data. MK and IL analyzed the raw data. MK made the statistical analysis and interpretation of the data. MK, IL, and AK wrote the article.

## Conflict of Interest Statement

The authors declare that the research was conducted in the absence of any commercial or financial relationships that could be construed as a potential conflict of interest. The reviewer, HT, and handling editor declared their shared affiliation, and the handling editor states that the process nevertheless met the standards of a fair and objective review.
